# Own Variety Bias

**DOI:** 10.1177/2041669515593018

**Published:** 2015-10-18

**Authors:** Marjoleine Sloos, Andrea Ariza García

**Affiliations:** Interacting Minds Centre, Aarhus University, Denmark; Department of Aesthetics and Communication, Aarhus University, Denmark

**Keywords:** dialect identification, biased perception, French, Canadian French

## Abstract

In a language identification task, native Belgian French and native Swiss French speakers identified French from France as their own variety. However, Canadian French was not subject to this bias. Canadian and French listeners didn’t claim a different variety as their own.

“Which language variety do you perceive?” This question, elicited to establish dialect categorization, boundaries, and continua (see Tamati, 2010 and Baker, Eddington, & Nay, 2009 and references cited there) is usually posed as a forced choice question: possible answers are already provided. The reason is obviously that open choice questions are felt to be too difficult (like in Baker et al., 2009, p. 50). An apparent caveat of this method is that a proportion of answers consist of a (wild) guess. So let us face the facts and see what respondents answer on the open question “which language variety do you perceive?”

We conducted an online survey consisting of small excerpts of 20 to 25 s (two or three adjacent sentences) of read speech of French from France and Canadian French. The fragments were selected from the online corpus *Phonologie du Français Contemporain* (Durand, Laks, & Lyche, 2002). The selected fragments consisted of recordings of 12 speakers from Canada (six from Quebec and six from Peace River [Alberta Province]) and 12 speakers from France (six from Paris and six from Dijon), equally balanced for gender and varied for age. We investigated the ability to identify French from France and Canadian French by native speakers of four different French nationalects (viz. French from France, Canadian French, Belgian French, and Swiss French). In addition, we asked the subjects to estimate the level of standardness of the speech fragments. Respondents were contacted via e-mail and social networks of the authors. In total, 70 responses were received, but eight respondents (all from France) did not complete the question about the geographic variety and were excluded from further analysis. Of the remaining 62 respondents, 16 were from France, 11 from Belgium, 10 from Canada, and 25 from Switzerland. The subjects were asked to estimate the level of standardness on a 5-point Likert scale as *standard*, *near-standard*, *a bit standard*, *nonstandard,* or *not at all standard*, and were asked to estimate the speaker’s location. They could listen to each fragment multiple times, go back and forth between the fragments, and adjust the volume to a limited extent.

The responses were divided into three main groups: correct, undecided, and incorrect. Correctness was evaluated at the level of the nationalect. Answers referring to the local level like “south of France” were counted as correct as long as they fell into the target nationalect. Undecided were all answers equivalent to “don’t know,” as well as answers that did not address the geographical variation but rather stylistic aspects, such as “very monotonous.” As expected, responses in the category “undecided” formed a large part of the total amount of responses: 35%. The remainder of this article is based on the total number of correct and incorrect answers, without taking into account the “undecided” responses.

Incorrect answers could be categorized into three subgroups:

1. identification of a variety as the respondent’s native variety, although it was not (i.e., own variety bias)
2. the identification of a variety other than the respondent’s native variety as second language accented French
3. the identification of the respondent’s native variety (i.e., French from France or Canada) as a different French variety.

We investigated the response patterns of the four listener groups (French, Canadian, Belgian, and Swiss) with a cluster analysis (Tagliamonte & Baayen, 2012), which is illustrated in Figure 1. The Belgian and Swiss listeners showed the same pattern and thus formed one group. We call this the outgroup, because their native variety was not included in the data. The main difference is found in the response patterns on the Canadian and French data (see Figure1, Node 1). Further, we observe a noticeable difference between the French and the Canadian respondents on the one hand and the outgroup respondents on the other hand: the French and the Canadian respondents only sporadically (3% and 2%, respectively) identified the other variety as one’s own, whereas the Belgian and Swiss respondents identified, respectively, 10% and 16% of the speech fragments as their own variety (see Figure 1, Nodes 6, 8 and 9). Besides, we observed that the Canadian listeners judged more French data incorrectly as a different French variety than the other participants (Node 9).

**Figure 1.**
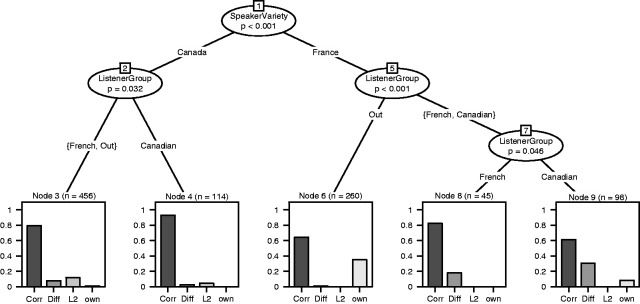
Clustering of the responses as correct (Corr), different French variety (Diff), French as a second language (L2), and own variety as an incorrect response (own).

As for own variety bias, the Belgian subjects claimed 17% of the fragments from Dijon and 8% from Paris as their own, and one answer expressed a doubt between French from France and Belgian French. The Swiss listeners, in total, claimed 23% of the fragments from Dijon, 19% from Paris, 1 from Quebec, and 2 from Peace River as their own, and in 3% of the cases indicated doubt between French from France and Swiss French (these cases were included as own variety bias). In four cases (3%), Swiss respondents explicitly and incorrectly claimed a Neuchatel accent, which was actually the variety of their own city of residence. Note that own variety bias does only occur in a significant proportion in the perception of French from France and not of Canadian French.

The differences in patterning across the French data as shown in Figure 1 are statistically significant. A logistic regression test (in R statistical environment [R Development Core Team, 2009]) with model comparison showed a significant interaction between outgroup (i.e., Belgian and Swiss) listeners and French as the speaker’s variety (*z* = 5.133, *p* < .001). We also found a significant interaction between Canadians as listener group and French as the speaker’s variety (*z* = 3.019, *p* = .003). The different pattern of the Canadian listeners in the Canadian data as compared with the patterns of the other groups was also significant (*z* = −2.919, *p* = .004). In addition, there was a correlation with estimated standardness such that the more standard the speech was perceived, the more likely it was that own variety bias occurred (*z* = 3.111, *p* = .002). All results are shown in Table 1.

**Table 1.** The Estimated, Standard Error, *z* Value, and *p* Value (Starred at the 95% Confidence Level) for Listener Group (With the Levels French, Canadian, and Outgroup [viz. Belgian and Swiss]) and the Speaker Variety (With the Levels French and Canada).

	Estimates	*SE*	*z*	*p*
Intercept	−2.417	0.407	−5.941	<.001*
Standardness	0.383	0.123	3.111	.002*
Listeners outgroup: speaker variety France	1.394	0.272	5.133	<.001*
Listeners Canadian: speaker variety France	1.370	0.454	3.019	.003*
Listeners Canadian: speaker variety Canada	−1.587	0.544	−2.919	.004*
Standardness	0.383	0.123	3.111	.002*

As far as we know, own variety bias in language identification has not been reported earlier as such. However, what scanty evidence is available shows that our findings don’t form an isolated case. For example, Boula de Mareuil and Vieru-Dimulescu (2006) showed that sentences with Spanish phonemes and Italian intonation as well as sentences with Italian phonemes and Spanish intonation were more likely to be judged as Italian with a Spanish accent by Italian listeners but as Spanish with an Italian accent by Spanish listeners. Further, Williams, Garrett, and Coupland (1999, p. 353) observed in an identification study of Welsh dialects among adolescents, that more than half of the Cardiff listeners identified Northwest Welsh as Cardiff, although these dialects are considerably distinct from each other. In a vowel perception study, Niedzielski (1999) showed that participants were likely to perceive vowels from a different variety as belonging to their own variety.

In conclusion, this article showed that Belgian French and Swiss French speakers identified a considerable portion of the fragments of French from France as their own variety. We believe this misidentification is grounded in their expectation that fragments of their variety were presented in the study, raised by the simple fact that the Belgian and Swiss subjects were asked to participate in the experiment. The Canadian fragments were likely to be so accented that they were not susceptible to own variety bias. Canadian and French participants are also likely to have had this expectation, but since their varieties were presented, and recognized, own variety bias didn’t reach the surface.
